# Accuracy of Estimating Periodontitis and Its Risk Association Using Partial-Mouth Recordings for Surveillance Studies: A Systematic Review and Meta-Analysis

**DOI:** 10.1155/2022/7961199

**Published:** 2022-03-17

**Authors:** Yasmine N. Alawaji, Abdulsalam Alshammari, Jolanta Aleksejuniene

**Affiliations:** ^1^Department of Preventive Dental Science, College of Dentistry, King Saud bin Abdul-Aziz University for Health Sciences, Riyadh, Saudi Arabia; ^2^King Abdullah International Medical Research Center, Riyadh, Saudi Arabia; ^3^Department of Oral Health Sciences, Faculty of Dentistry, The University of British Columbia, Vancouver, Canada

## Abstract

**Objectives:**

Our aim is to conduct an up-to-date systematic review and meta-analysis pertaining to the accuracy of using the partial-mouth recording protocol (PRP) in surveillance studies to estimate the periodontitis prevalence, extent, severity, and its risk associations.

**Methods:**

Medline and Embase databases were searched for studies which assessed the periodontitis prevalence, severity, extent, or its risk associations using PRPs versus full-mouth recording protocols (FRPs); searches were conducted up until May 26, 2021. The risk of bias and the applicability of the studies were assessed using the QUADAS-2 tool. Both qualitative data synthesis and quantitative data synthesis were performed, and comparisons were done for the accuracy and precision of PRPs for different periodontitis outcomes. The study's protocol was registered through the International Platform of Registered Systematic Review and Meta-analysis Protocols (registration number: INPLASY202160032).

**Results:**

A total of 14 studies were included. The studies had a considerable degree of heterogeneity, along with a moderate risk of bias and applicability concerns. Several factors influenced the accuracy or precision of using PRPs, including the age, distribution of periodontitis in the studied population, PRP selection, total PRP sites, the threshold for minimum sites with CAL, and the severity of periodontitis case definitions. Overall, the PRP with the highest accuracy and precision mainly included (1) a full-mouth protocol at the following partial sites: mesiobuccal-midbuccal-distolingual (MB-B-DL), mesiobuccal-distolingual (MB-DL), mesiobuccal-midbuccal-distobuccal (MB-B-DB), mesiobuccal-distobuccal (MB-DB), and 84 sites using the random site selection method (RSSM) and (2) random-half-mouth (RHM) protocols.

**Conclusions:**

The PRPs with the highest overall accuracy and precision in estimating the periodontitis prevalence, extent, severity, and risk associations included the full-mouth assessment at the following partial sites: MB-B-DL, MB-DL, MB-B-DB, MB-DB, and 84 sites using RSSM and RHM protocols.

## 1. Introduction

Epidemiological studies mainly focus on the assessment of certain disease distributions in populations and their risks [1]. A full-mouth recording protocol (FRP) examines six sites per tooth in all teeth except for the third molars and is considered the gold standard for periodontal examinations [2, 3]. However, due to the extensive time, cost, and the number of examiners needed to conduct population-based studies, the use of a partial-mouth recording protocol (PRP) can be an alternative to FRP [2].

Several PRPs were tested for accuracy, including the protocol examining index teeth such as the Ramfjord teeth and Community Periodontal Index of Treatment Needs (CPITN), the random-half-mouth (RHM) protocol, and fixed or random selection of partial site protocols [4–8]. In a 2013 systematic review [9], various PRPs were tested for their accuracy and found that the highest accuracy when examining the prevalence, severity, and extent of periodontitis resulted from using the RHM protocol at six sites per tooth and full-mouth recordings at Mesiobuccal-Midbuccal-Distobuccal sites [(FM)MB-B-DB]. Conducting half-mouth examinations at the Mesiobuccal-Midbuccal-Distolingual [(HM)MB-B-DL] or (FM)MB-B-DL sites were also effective in assessing the prevalence and severity of periodontitis. However, the (HM)MB-B-DL or (FM)MB-B-DL protocols were not used to evaluate the extent of periodontitis.

Previous studies that evaluated the accuracy of PRPs mainly focused on examining periodontitis prevalence and/or summarizing its extent and severity [4, 7, 9–12]. Only recently have studies started to assess the precision of using PRPs to estimate the risks associated with periodontitis [13, 14]. A previous 2013 systematic review assessed the validity of PRPs for studying periodontitis severity, extent, and prevalence using a single disease threshold, and the precision of using PRPs to assess the risk associated with periodontitis was not evaluated. In addition, the factors that could impact the accuracy or precision of PRPs were not addressed in the previous systematic review; therefore, the evidence needs to be updated [15].

The main research question was “Should we rely on PRP for assessment of periodontitis in surveillance surveys?” There were two specific objectives in this study: (1) to conduct an up-to-date systematic review and meta-analysis regarding the accuracy of PRPs to estimate periodontitis prevalence, extent, severity, and its risk associations, and (2) to identify the factors that may impact the performance of PRPs.

## 2. Methods

This review was prepared using the Preferred Reporting Items for Systematic Review and Meta-analysis of Diagnostic Test Accuracy Studies (PRISMA-DTA) [16]. The study's protocol was registered in the International Platform of Registered Systematic Review and Meta-analysis Protocols (Registration number: INPLASY202160032) [17].

### 2.1. Eligibility

The following inclusion criteria were used: full-text papers in English about studies that employed a cross-sectional study design or that analyzed the baseline data of longitudinal studies, including subjects of any age with permanent dentitions, where all the PRP findings were verified with the FRP. FRP data assessed six sites or four interproximal sites per tooth in all teeth, except third molars. The studies reported the following outcomes: periodontitis prevalence, its risk associations, mean, and standard deviation (SD) for estimates of severity or extent. Studies used clinical attachment loss (CAL) since CAL is an irreversible indicator of periodontitis. The exclusion criteria are as follows if the studies included any of the following: a simulation study, hypothetical data, subjects with primary dentition, studies using other periodontal parameters such as the periodontal pocket depth (PPD), or bleeding upon probing without assessment of CAL.

### 2.2. Search Strategy

The search was conducted up until May 26, 2021, using keywords and MeSH or Emtree terms based on several field searches including titles, abstracts, and author keywords. The following search concepts were considered: periodontitis, prevalence, extent, severity, and partial-mouth recording. Limits or filters were not used when conducting the search to increase the search sensitivity. Medline and Embase were mainly searched via Ovid, and grey literature was searched at different sources as outlined in the protocol [17]. Manual searches were done for reference lists and related citations in PubMed.

### 2.3. Outcomes

There exist accuracy and precision of each of the following: (1) periodontitis prevalence as indicated by the measures of sensitivity, specificity, positive predictive value (PPV), Negative Predictive Value (NPV), and absolute bias; (2) periodontitis-related risk associations indicated by absolute bias and/or relative bias; (3) extent of periodontitis indicated by absolute bias; and (4) severity of periodontitis indicated by absolute bias.

Prevalence of periodontitis was defined using the two most commonly used thresholds in the previous studies: (1) severe periodontitis (≥1 site with CAL ≥6 mm) and (2) moderate-severe periodontitis (≥1 site with CAL ≥4 mm). Suboutcomes for periodontitis prevalence were defined using the case definitions from the Centers for Disease Control and Prevention and the American Academy of Periodontology (CDC/AAP) [18, 19]. The CDC/AAP severe periodontitis was defined as CAL ≥6 mm at ≥2 interproximal sites (not on the same tooth) and PPD ≥5 mm at ≥1 interproximal site (can be one of the two sites with CAL). Moderate-severe periodontitis was defined as CAL ≥4 mm at ≥2 interproximal sites (not on the same tooth) or PPD ≥5 mm at ≥2 interproximal sites (not on the same tooth).

Periodontitis extent was defined as the mean percentage of sites with CAL ≥3 mm or ≥5 mm [2]. Periodontitis severity was defined as the population mean of CAL. Absolute bias was calculated for each of the periodontal outcomes as follows:

- Absolute bias_prevalence_ = prevalence _PRP_ − prevalence _FRP_
- Absolute bias_extent_ = mean extent _PRP_ − mean extent _FRP_
- Absolute bias_severity_ = mean CAL _PRP_ − mean CAL _FRP_

In order to estimate the potential for systematic error concerning the risk associations with the periodontal disease when comparing PRPs to FRPs, the absolute bias and relative bias were calculated based on the natural logarithm (ln) scale of odds ratios (OR) as follows [13, 14]:

- Absolute bias of OR = ln (OR_PRP_) − ln (OR_FRP_)
- Relative bias of OR = [ln (OR_PRP_) − ln (OR_FRP_)]/ln (OR_FRP_)

### 2.4. Study Selection and Data Extraction

The preliminary study selection was done by screening the titles and abstracts, and the final selection was based on eligibility criteria after retrieval of the full texts. A single data selection and extraction were completed and then verified by another reviewer. Any disagreements in the study selection or data extraction were resolved by discussion until a consensus was reached. A data extraction form was customized using Microsoft Excel software, version 16.0 [20]. The following information about each extracted study was collected: author names, publication date, study title, sample size, study design, study settings/country, included age range, eligibility criteria, examiner training/calibration, intraexaminer/interexaminer reliability, management of missing data, the minimum number of included sites/teeth, PRP type, definition of FRP, periodontitis thresholds, and mean results of periodontitis extent and severity using the PRP and FRP. Total subjects with or without periodontitis in the PRP and FRP were used to construct a 2 × 2 table to calculate the prevalence of periodontitis using the following diagnostic accuracy indicators: sensitivity, specificity, and predictive values. For each periodontitis-associated risk such as diabetes mellitus, obesity, and cigarette smoking, both unadjusted and adjusted OR were extracted along with their 95% CI.

### 2.5. Assessment of Risk of Bias and the Applicability of Individual Studies

The second version of the Quality Assessment Tool for Diagnostic Accuracy Studies (QUADAS)-2 tool [21] was used for the assessment of the risk of bias (4 domains) and the applicability concerns (3 domains). Based on the suggestions by QUADAS-2, the signaling questions for each domain were customized for the current review, and their revised versions are presented in a supplementary table (Table S-1). For the overall judgment, a high risk of bias or high applicability concerns were determined if the study was rated as such in at least one of the domains. The customization of the tool and the risk of bias assessment were done by two reviewers, and any disagreement was resolved by discussion until a consensus was reached.

### 2.6. Data Synthesis

We used OpenMeta-Analyst software [22] to analyze data extracted from eligible studies to summarize and compare different accuracy and precision indicators for the prevalence, extent, and severity of periodontitis using different PRPs. Meta-analysis was used to compare the different PRPs and study methods rather than relying on the pooled estimates. The statistical heterogeneity was assessed as outlined in the Cochrane handbook [23] using the following assessments: (1) visual examination of the forest plots, where minimal or no overlap of studies' 95% confidence intervals and/or the variation of bias direction (positive or negative) indicates heterogeneity; (2) a Chi-square test where *α* = 0.100 indicates a statistical significance; and (3) *I*^2^ used to quantify the heterogeneity, where *I*^2^ range from 0.0% to 40.0% indicates negligible heterogeneity, *I*^2^ ≥ 75% indicates a considerable heterogeneity, and *I*^2^ values between 40.0% and 75.0% indicate moderate to substantial heterogeneity. For the meta-analysis, we chose a random effect model (DerSimonian and Laird inverse variance) for pooling the results of different PRPs. In order to identify sources of heterogeneity and to identify the factors that impact the performance of PRPs, the clinical and methodological diversity of the included studies were assessed. Subanalyses were done for estimating the prevalence of sensitivity and absolute bias of studies that used case definitions provided by the CDC/AAP.

## 3. Results

### 3.1. Study Selection

**Figure 1:**
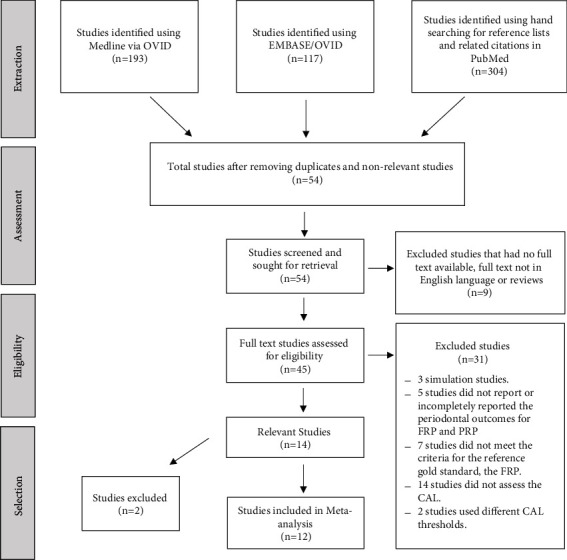
Flow chart of study selection, data extraction, and analysis. *n*: total number of studies, FRP: full-mouth recording rrotocol, PRP: partial-mouth recording protocol, CAL: clinical attachment loss, and CPITN: community periodontal index of treatment needs.

A total of 614 papers were retrieved for preliminary screening of titles and abstracts. Total numbers of included and excluded studies are presented in Figure 1. A detailed explanation of the reasons for exclusion is presented in the appendix (Table A-1). The main types of PRPs in the included studies were the full mouth at partial sites, RHM, half mouth at partial sites, and index teeth. The risk of bias and applicability assessment of 14 selected studies are presented in Table 1. A summary of study characteristics and potential concerns are described in Table 2. Most of the studies did not describe how the sample size was calculated or how they handled the missing data. A majority of studies limited the inclusion of subjects if they had ≥6 teeth remaining. Few studies had strict eligibility criteria such as subjects being required to have ≥16–24 teeth present [24–26], limited to subjects with periodontitis [25], or untreated individuals [27, 28], which can limit their external validity.

### 3.2. Prevalence of Periodontitis

**Figure 2:**
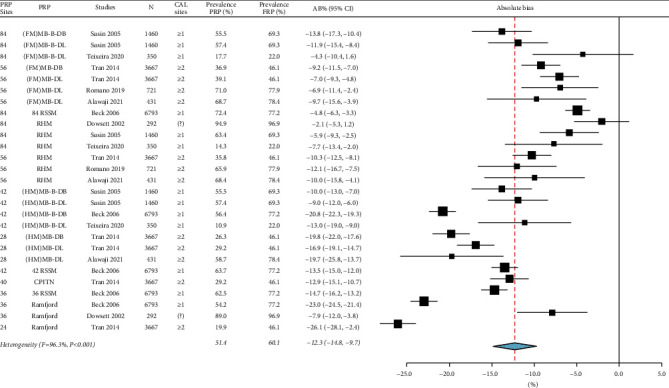
Summary of the absolute bias (AB)_prevalence_ of moderate-severe periodontitis using partial-mouth recording protocols (PRP). Total PRP sites for each PRP and the minimal number of sites with clinical attachment loss (CAL) are listed. AB_prevalence_ values <0.0 underestimate the prevalence while values >0.0 overestimate it. FRP: full-mouth recording protocol, N: sample size, CI: confidence interval, MB: mesiobuccal, B: Midbuccal, DB: distobuccal, ML: mesiolingual, L: midlingual, DL: distolingual, FM: full-mouth, HM: half-mouth, 84 RSSM: 84 sites selected using random site selection method, 42 RSSM: 42 sites selected using random site selection method, 36 RSSM: 36 sites selected using random site selection method, (RHM): random-half-mouth, CPITN: Community Periodontal Index of Treatment Needs, and ?: not clear.

**Figure 3:**
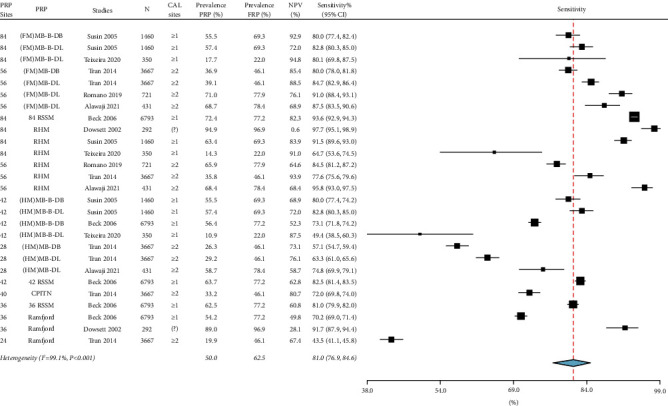
Summary of the sensitivity of moderate-severe periodontitis prevalence using partial-mouth recording protocols (PRP). Specificity and positive predictive value are 100% for all PRPs. PRP sites, the minimal number of sites with clinical attachment loss (CAL), and negative predictive value (NPV) are listed. FRP: full-mouth recording protocol, N: sample size, CI: confidence interval, MB: mesiobuccal, B: midbuccal, DB: distobuccal, ML: mesiolingual, L: midlingual, DL: distolingual, FM: full-mouth, HM: half-mouth, 84 RSSM: 84 sites selected using random site selection method, 42 RSSM: 42 sites selected using random site selection method, 36 RSSM: 36 sites selected using random site selection method, RHM: random-half-mouth, CPITN: Community Periodontal Index of Treatment Needs, and ?: not clear.

**Figure 4:**
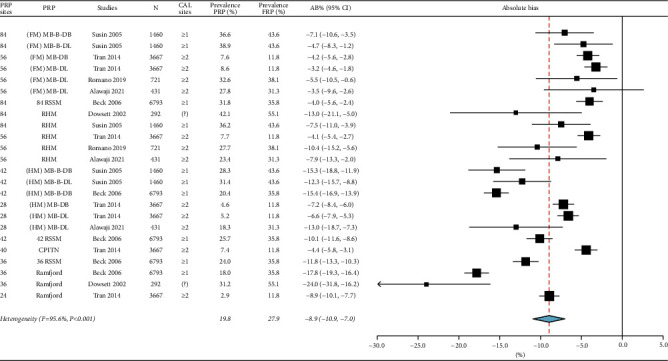
Summary of the absolute bias (AB)_prevalence_ of severe periodontitis using partial-mouth recording protocols (PRP). Total PRP sites for each PRP and the minimal number of sites with clinical attachment loss (CAL) are listed. AB_prevalence_ values <0.0 underestimate the prevalence while values >0.0 overestimate it. FRP: full-mouth recording protocol, N: sample size, CI: confidence interval, MB: mesiobuccal, B: midbuccal, DB: distobuccal, ML: mesiolingual, L: midlingual, DL: distolingual, FM: full-mouth, HM: half-mouth, 84 RSSM: 84 sites selected using random site selection method, 42 RSSM: 42 sites selected using random site selection method, 36 RSSM: 36 sites selected using random site selection method, RHM: random-half-mouth, CPITN: Community Periodontal Index of Treatment Needs, and ?: not clear.

**Figure 5:**
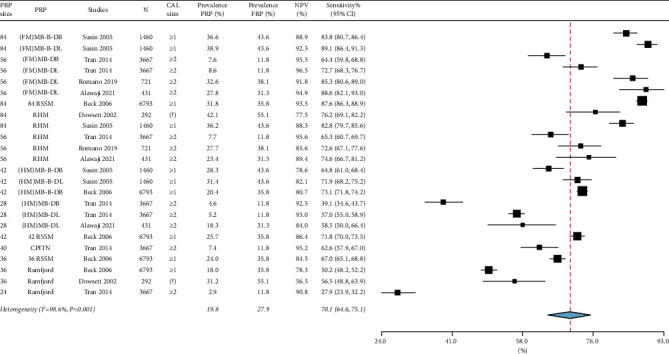
Summary of the sensitivity of severe periodontitis prevalence using partial-mouth recording protocols (PRP). Specificity and positive predictive value are 100% for all PRPs. PRP sites, the minimal number of sites with clinical attachment loss (CAL), and negative predictive value (NPV) are listed. FRP: full-mouth recording protocol, N: sample size, CI: confidence interval, MB: mesiobuccal, B: midbuccal, DB: distobuccal, ML: mesiolingual, L: midlingual, DL: distolingual, FM: full-mouth, HM: half-mouth, 84 RSSM: 84 sites selected using random site selection method, 42 RSSM: 42 sites selected using random site selection method, 36 RSSM: 36 sites selected using random site selection method, RHM: random-half-mouth, CPITN: Community Periodontal Index of Treatment Needs, and ?: not clear.

For moderate-severe periodontitis, the prevalence ranged from 22.0% to 96.9%, the absolute bias_prevalence_ was −12.3% [(95% CI: −14.8%, −9.7%), *I*^2^ = 96.3%, *p* < 0.001] in Figure 2, and the sensitivity was 81.0% [(95% CI: 76.9%, 84.6%), *I*^2^ = 99.1%, *p* < 0.001] in Figure 3. For severe periodontitis, the prevalence ranged from 11.8% to 55.1%, the absolute bias_prevalence_ was −8.9% [(95% CI: −10.9%, −7.0%), *I*^2^ = 95.6%, *p* < 0.001] in Figure 4, and the sensitivity was 70.1% [(95% CI: 64.6%, 75.1%), *I*^2^ = 98.6%, *p* < 0.001] in Figure 5. Moderate-severe periodontitis had larger absolute bias_prevalence_ (−12.3% versus −8.9%), higher sensitivity (81.0% versus 70.1%), and lower overall NPV compared with severe periodontitis (Figures 2–5). In a subanalysis of studies that used the CDC/AAP definitions for periodontitis, a similar pattern was seen when absolute bias, sensitivity, and NPV measures were compared between moderate-severe and severe periodontitis thresholds (refer to supplementary Figures S-1–S-4).

For both periodontitis thresholds, the heterogeneity among studies was considerable for the absolute bias_prevalence_ and sensitivity as indicated by the *I*^2^ > 95.0 and significant Chi-square results (*p* < 0.001) (Figures 2–5). Sources of heterogeneity and the factors that may impact the performance of PRPs include prevalence and severity of periodontitis case definition, using CAL measurements alone or in combination with PPD in case definitions, different minimal number of sites to used define periodontitis (≥1 or ≥2 sites) as presented in Table 3, the PRP type, and the total PRP sites (some studies used the midbuccal and midlingual sites, while others included only interproximal sites).

### 3.3. Estimate of Periodontitis Severity

**Figure 6:**
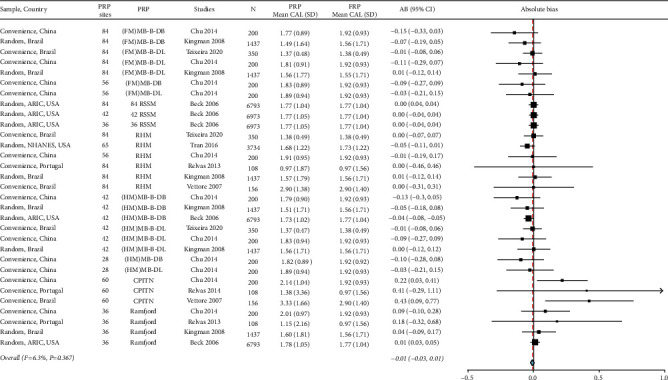
Summary of absolute bias (AB)_severity_, background characteristics of studies, PRP sites, and severity estimates are listed. FRP: full-mouth recording, SD: standard deviation, N: sample size, CI: confidence interval, NHANES: National Health and Nutrition Examination Survey, ARIC: atherosclerosis risk in communities, MB: mesiobuccal, B: midbuccal, DB: distobuccal, ML: mesiolingual, L: midlingual, DL: distolingual, FM: full-mouth, 84 RSSM: 84 sites selected using random site selection method, 42 RSSM: 42 sites selected using random site selection method, 36 RSSM: 36 sites selected using random site selection method, HM: Half-mouth, and CPITN: Community Periodontal Index of Treatment Needs.

The absolute bias_severity_ was −0.01 (95% CI: −0.03, 0.01) which is considered small and the majority of PRPs had their 95% CI including the null value. The use of index teeth: Ramfjord teeth, and CPITN protocols mainly overestimated the severity, and the largest absolute bias_severity_ was 0.4. The heterogeneity for absolute bias_severity_, as indicated by *I*^2^ = 6.25 and a chi-square test *p*=0.367 (Figure 6), can be considered negligible [23].

### 3.4. Estimate of the Periodontitis Extent

**Figure 7:**
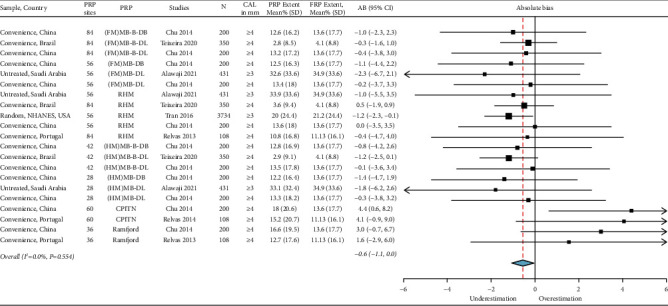
Summary estimates of the absolute bias (AB)_extent_ of moderate-severe periodontitis. Background characteristics of studies, PRP sites, and specific thresholds of clinical attachment loss (CAL) are listed. FRP: full-mouth recording protocol, N: sample size, SD: standard deviation, CI: confidence interval, NHANES: national health and nutrition examination survey, MB: mesiobuccal, B: midbuccal, DB: distobuccal, ML: mesiolingual, L: midlingual, DL: distolingual, FM: full-mouth, HM: half-mouth, and CPITN: Community Periodontal Index of Treatment Needs.

**Figure 8:**
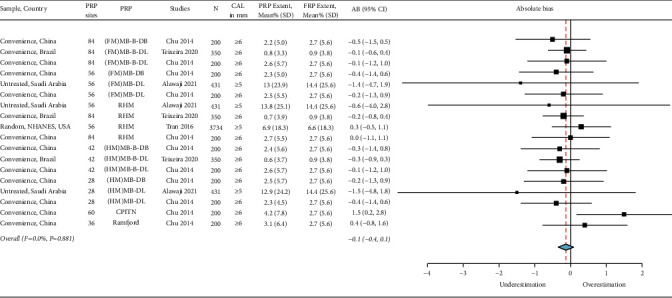
Summary of the absolute bias (AB)_extent_ of severe periodontitis using partial-mouth recording protocols (PRP). Total PRP sites for each PRP are listed. AB_extent_ values <0.0 underestimate the extent, while values >0.0 overestimate it. FRP: full-mouth recording protocol, N: sample size, SD: standard deviation, CI: confidence interval, MB: mesiobuccal, B: midbuccal, DB: distobuccal, ML: mesiolingual, L: midlingual, DL: distolingual, FM: full-mouth, HM: half-mouth, 84 RSSM: 84 sites selected using random site selection method, 42 RSSM: 42 sites selected using random site selection method, 36 RSSM: 36 sites selected using random site selection method, RHM: random-half-mouth, and CPITN: Community Periodontal Index of Treatment Needs.

The absolute bias_extent_ was −0.6 [(95% CI: −1.1, 0.0), *I*^2^ = 0.00%, *p*=0.554] for moderate-severe periodontitis (Figure 7) and −0.1 [(95% CI: −0.4, 0.1), *I*^2^ = 0.0%, *p*=0.881] for severe periodontitis (Figure 8). The extent of periodontitis was mainly underestimated when using PRPs; however, the use of index teeth: Ramfjord teeth, and CPITN protocols overestimated the periodontitis extent. The overall heterogeneity for moderate-severe and severe periodontitis was negligible as indicated by the *I*^2^ = 0.0 and the nonsignificant Chi-square findings for moderate-severe and severe periodontitis (Figures 7 and 8).

### 3.5. Periodontitis-Related Risk Associations

A few studies assessed the accuracy of the risk associations of periodontitis using PRPs compared with FRP, which precluded us from conducting a meta-analysis. We chose to summarize the risk determinants that were reported in two or more studies: diabetes mellitus, obesity, and current cigarette smoking status (Table 4). The measurement of these risk determinants varied among studies. Diabetes mellitus was either defined as present/absent [13, 14] or based on self-reported glycemic control [28]. Obesity was defined as present/absent [28] or based on Body Mass Index [13, 14]. Current smoking was defined as currently smoking any number of cigarettes daily [28] or smoking ≥100 cigarettes during the lifetime [13].

## 4. Discussion

The current systematic review and meta-analyses aimed to answer the research question “should we rely on PRPs to study periodontal diseases in surveillance studies?”. The two main objectives were to examine the accuracy and precision of determining the prevalence, severity, extent, and risk associations of periodontitis using PRP as compared to FRP and to identify the factors that may influence accuracy using PRP.

### 4.1. Prevalence of Periodontitis

Specificity and positive predictive values were 100.0% for all PRPs because PRPs can only identify positive cases that were already identified using an FRP [32]. The average sensitivity was 81.0% for moderate-severe periodontitis (CAL ≥4 mm), which can be considered a high sensitivity [33]. The sensitivity measures of individual PRPs for moderate-severe periodontitis were mainly above 80.0% for the RHM and full-mouth assessments at partial sites such as the (FM)MB-B-DL, MB-DL, MB-B-DB, MB-DB, and 84, 42, and 36 RSSM. The NPV varied considerably among different studies and ranged from as low as 0.6% to 94.8%, which highlights the fact that the accuracy of PRPs can be influenced by the prevalence of periodontitis in specific populations [34].

When the periodontitis threshold increased from moderate (CAL ≥4 mm) to severe (CAL ≥6 mm), the average sensitivity decreased to 70.1%, which is considered only a moderate sensitivity [33]. The PRPs that resulted in sensitivity above 70.0% were the RHM and full-mouth assessments at partial sites such as (FM)MB-B-DL, MB-DL, MB-B-DB, MB-DB, 84, 42, and 36 RSSM.

The minimum number of sites with CAL used in the included studies was either ≥1 or ≥2 sites. A higher sensitivity from 7.4 to 21.8% resulted when CAL was assessed at ≥1 site compared to CAL at ≥2 sites. However, the use of CAL measurements at ≥2 sites is preferable in order to preclude sites with CAL due to other etiologies such as having a single isolated PPD due to endodontic involvements or root fractures.

### 4.2. Periodontitis Severity and Extent

In general, absolute bias and heterogeneity were low across different studies, PRPs, and CAL thresholds. The index teeth, Ramfjord teeth, and CPITN teeth protocols overestimated the extent and severity of periodontitis and, thus, resulted in the highest absolute bias.

### 4.3. Risk Associations with Periodontitis

The three risk determinants summarized in this study were diabetes mellitus, obesity, and cigarette smoking. In general, the absolute bias was small and ranged from −0.5 to 0.9. This means that the use of PRP can marginally underestimate or overestimate the risk associations with periodontitis. However, the evidence was limited for several reasons. Until now, only a few studies evaluated the precision of using PRPs to assess the risk determinants of periodontitis compared to FRPs. Importantly, the risk determinants depend to some degree on the prevalence of periodontitis and the use of adjusted or nonadjusted odds ratios. Also, the size of the adjusted risk association will change based on other variables included in the multivariate models. In addition, the definitions and the measurements of the risk associations varied among studies. Diabetes mellitus was defined as present/absent in two of the included studies [13, 14]. However, based on current knowledge, a lack of glycemic control is more important than the presence or absence of diabetes mellitus because diabetic subjects with good glycemic control (e.g., HbA1c <7%) have a similar risk for periodontal disease as nondiabetic individuals [35–37]. Therefore, we recommend using objective measurements such as the glycemic control of individuals when studying periodontitis-related risk associations.

The majority of studies included a wide age range of subjects (13–103 years). However, a limited number of studies reported the comparisons based on age cohorts. Two studies analyzed the precision of estimating periodontitis extent and/or severity and did not find clear patterns based on age cohorts [9, 27]. One study [28] compared subjects 13–34 years of age to an older group 35–80 years of age and found that the accuracy of estimating the prevalence of periodontitis was lower in younger subjects, while the extent of periodontitis was either underestimated or overestimated; this is consistent with previous findings [38].

### 4.4. Review Limitations and Implications

The majority of the studies used nonrepresentative and nonrandomly selected samples [24–29] or randomly selected subjects without reporting the extent of the missing data [4, 7–10, 13, 14, 39]. In addition, some studies restricted their samples to subjects with periodontitis [25] and untreated subjects [27, 28], or they excluded subjects with 14 to 23 teeth remaining [24–26]. Studies that limited their assessment of periodontitis to measuring the PPD without assessment of CAL were excluded. The use of PPD alone can be a reversible indicator of periodontitis; i.e., periodontal treatment may reduce the PPD and thus underestimate the periodontitis prevalence [40, 41].

Most of the studies in the literature compared the performance of PRPs to FRPs in terms of accuracy, validity, and/or precision. However, there is no published evidence that compares the actual time, effort, and resources needed to conduct any of the PRPs compared to the FRPs. Also, the feasibility of applying some of the PRPs, such as the RSSM at 36, 42, or 84 sites, can be challenging in surveillance studies [4].

### 4.5. Review Implications

Including a wide age range, four periodontitis outcomes, and several disease thresholds make our findings more generalizable. We investigated the methodological factors that may impact the performance of PRPs which can guide the selection of protocols in future surveillance studies. In addition, our review highlights some of the limitations in the literature and methodological considerations that need to be addressed in future studies.

### 4.6. Future Directions

Some of the recent studies [42, 43], despite the fact that they were excluded due to the deficiency of their clinical data, have used promising approaches for the assessment of optimal PRP selection and the optimal case definitions. The optimal selection of PRPs was tested using the item response theory model, where they aimed to select a PRP that examines the least number of sites while retaining the highest accuracy compared to an FRP [43]. This study suggests using a PRP with only 12 sites, which is one of the PRPs with the least number of sites while still providing high accuracy; however, further testing of this approach and its feasibility is recommended. For the selection of the optimal periodontal disease thresholds, the receiver operating characteristic curve and the area under the curve should be used to evaluate the selected disease thresholds versus the optimal disease thresholds using PRPs rather than FRPs [42].

We reiterate the following recommendations for the use of PRPs for surveillance studies.

Before conducting a national survey using a PRP, pilot studies should first be conducted in a subset sample of a specific population with an adequate representation of subgroups [2, 8]. In these pilot studies, several accuracy measures should be used, as well as absolute bias and relative bias. If necessary, a correction factor for the PRP can be calculated to acquire valid estimates of disease prevalence in a specific population [7, 44].

The use of CAL measurements at ≥1 interproximal sites to define the periodontal disease prevalence increased the sensitivity and slightly decreased the specificity. However, the use of these measurements at ≥2 interproximal sites is preferable to exclude sites with CAL due to other etiologies, such as trauma from brushing, orthodontic treatments that may result in midbuccal or midlingual CAL, isolated sites with endodontic involvements, vertical root fractures, and external root resorption.

Objective measurements of medical conditions, such as an assessment of glycemic control using HbA1c, are needed to confirm the risk associated with diabetes mellitus. Also, both adjusted and unadjusted effects of risk determinants should be presented in order to compare the results of different studies.

## 5. Conclusions

Several factors influenced the accuracy and precision of PRPs and need to be considered before conducting surveillance studies; these include the age, prevalence, and severity of periodontitis in a specific population, type of PRP, total PRP sites, and use of a minimum number of sites with CAL in periodontitis case definitions. Based on our results, the PRPs with the highest accuracy and precision to assess periodontitis prevalence included full-mouth recordings at the following partial sites: MB-B-DL, MB-DL, MB-B-DB, MB-DB, 84 randomly selected sites (84 RSSM), and the RHM protocol at 6 sites per tooth (or 4 interproximal sites). Overall, the PRPs estimated the periodontitis extent and severity with a relatively high degree of precision. Risk factors associated with periodontitis resulted in only marginal information loss, regardless of the type of the PRP; however, this evidence is based on a small number of studies.

## Figures and Tables

**Table 1:** Quality assessment of the included studies using the second version of quality assessment of diagnostic accuracy studies tool (QUADAS-2).

Authors, date	Patient selection		PRP		FRP		Flow	Overall ROB	Overall applicability concerns
	ROB	Applicability concerns	ROB	Applicability concerns	ROB	Applicability concerns	ROB		

Alawaji et al., 2021	Low	Unclear	Low	Low	Low	Low	Low	Low risk	Potential concerns
Teixeira et al., 2020	Unclear	Unclear	Low	Low	Low	Low	Low	Potential risk	Potential concerns
Alshihayb et al., 2020	Low	Low	Low	Low	Low	Low	Low	Low risk	Low concerns
Romano et al., 2019	Low	Low	Low	Low	Low	Low	Low	Low risk	Low concerns
Tran et al., 2016	Low	Low	Low	Low	Low	Low	Low	Low risk	Low concerns
Akinkugbe et al., 2015	Unclear	Unclear	Low	Low	Low	Low	Low	Potential risk	Potential concerns
Tran et al., 2014	Low	Low	Low	Low	Low	Low	Low	Low risk	Low concerns
Chu and Ouyang, 2014	High	High	Low	Low	Low	Low	Low	High risk	High concerns
Relvas et al., 2013	Low	High	Low	Low	Low	Low	Low	Low risk	High concerns
Kingman et al., 2008	Low	Low	Low	Low	Low	Low	Low	Low risk	Low concerns
Vettore et al., 2007	Low	High	Low	Low	Low	Low	Low	Low risk	High concerns
Beck et al., 2006	Low	Unclear	Low	Low	Low	Low	Low	Low risk	Potential concerns
Susin et al., 2005	Low	Unclear	Unclear	Low	Low	Low	Low	Potential risk	Potential concerns
Dowsett et al., 2002	Unclear	Unclear	Unclear	Low	Low	Low	Unclear	Potential risk	Potential concerns

ROB: risk of bias, PRP: partial-mouth recording protocol, and FRP: full-mouth recording protocol.

**Table 2:** Included studies' characteristics and potential concerns.

Study author, date, citation	Settings, country	Sample size, age range	Outcomes	Partial recording protocol (PRP)	Minimum number of teeth or sites	Potential concerns
Alawaji et al., 2021	Untreated individuals at a university setting, Saudi Arabia	431 subjects, 13–80 years	Prevalence of CDC/AAP moderate-severe and severe periodontitis, mean (SD) of periodontitis extent, risk associations including self-reported diabetes mellitus, obesity, and current cigarette smoking	(FM)MB-DL, RHM, and (HM)MB-DL	3 teeth	Targeted untreated subjects recruited at university settings which may limit the external validity. The medical conditions were self-reported by the subjects without adding objective measurement for glycemic control, obesity was self-reported as present/absent, no minimum number of cigarette smoking were included in the current smokers' group
Teixeira et al., 2020	Convenience sample, Brazil	350 subjects, 35–74 years	Prevalence of moderate periodontitis, mean (SD) of periodontitis severity, and extent	(FM)MB-B-DL, RHM, and (HM)MB-B-DL	4 teeth	Did not describe the background characteristics of the study subjects. It was not clear where the clinical examinations were conducted
Alshihayb et al., 2020	General population (NHANES 2009–2014), USA	9575 subjects, 30–79 years	Risk associations to periodontitis prevalence including self-reported diabetes mellitus	RHM, CPITN teeth, and Ramfjord teeth	2 teeth for each used PRP	Did not define the eligibility criteria for included subjects from the NHANES data, excluded all subjects with missing data, did not report the values for the dependent variables used in the multivariate models. Defined diabetes mellitus status as present or absent without considering glycemic control
Romano et al., 2019	General population, Italy	721 subjects, 20–75 years	Prevalence of CDC/AAP moderate-severe and severe periodontitis	(FM)MB-B-DL and RHM	6 teeth	Excluded subjects with less than 6 remaining teeth
Tran et al., 2016	NHANES 2009–2010, USA	3734 subjects, 30–80 years	Mean (SD) of periodontitis extent and severity	RHM	1 tooth per selected quadrant	Excluded subjects who had no teeth in the selected quadrants for the RHM
Akinkugbe et al., 2015	General population based (ARIC study), USA	6259 subjects, 45–64 years	Risk associations were listed including current smoking (total of ≥100 cigarettes in a lifetime) and diabetes mellitus	42 RSSM, (HM)MB-B-DL, and Ramfjord teeth	2 eligible sites	Eligibility criteria were not described, excluded subjects who had 1 site with CAL rather than considering them in the no periodontitis category. Defined diabetes mellitus status as present or absent without considering glycemic control
Tran et al., 2014	General population (NHANES data 2009–2010), USA	3667 subjects, 30–80 years	Prevalence of CDC/AAP moderate-severe and severe periodontitis	(FM)MB-DB, (FM)MB-DL, RHM, (HM)MB-DL, (HM)MB-DL, CPITN teeth, and Ramfjord teeth	6 teeth	Excluded all subjects with missing data and those who had less than 6 remaining teeth
Chu and Ouyang, 2014	Convenience sample, China	200 subjects, 22–64 years	Mean (SD) of periodontitis severity and extent	(FM)MB-B-DB, (FM)MB-B-DL, (FM)MB-DB, (FM)MB-DL, RHM, (HM)MB-B-DB, (HM)MB-B-DL, (HM)MB-DB, (HM)MB-DL, Ramfjord teeth, and CPITN teeth	≥16 remaining teeth, ≥4 of them are molars, having ≥1 site with ≥5 mm PPD and CAL ≥2 mm in ≥2 sites in different quadrants	Convenience sample, selected subjects with ≥16 remaining teeth with periodontitis which limit the external validity
Relvas et al., 2013	Convenience sample, Portugal	108 subjects, 25–65 years	Mean (SD) of periodontitis severity and extent	RHM, Ramfjord teeth, and CPITN teeth	≥24 teeth, ≥5 teeth per quadrant, ≥8 teeth in CPITN, ≥4 teeth in Ramfjord	Convenience sample and strict eligibility criteria that limits the external validity of the study
Kingman et al., 2008	Population based, Brazil	1437 subjects, 14–103 years	Mean (SD) of periodontitis severity	(FM)MB-B-DB, (FM)MB-B-DL, RHM, (HM)MB-B-DB, (HM)MB-B-DL, and Ramfjord teeth	≥6 teeth per quadrant	Minimum number of included teeth may limit the external validity of the study
Vettore et al., 2007	University setting, Maternity clinic, Brazil	156 subjects, 30–67 years	Mean (SD) of periodontitis severity	RHM and CPITN	≥15 teeth	Minimum number of included teeth may limit the external validity
Beck et al., 2006	Population based (ARIC study), 4 states, USA	6793 subjects, 45–64 years	Prevalence of moderate-severe and severe periodontitis, mean (SD) of periodontitis severity	84 RSSM, 42 RSSM, 36 RSSM, RHM, (HM)MB-B-DB, and Ramfjord teeth	≥1 tooth	Older subjects (52–74 years), which may limit the external validity
Susin et al., 2005	General population, Brazil	1460 subjects, 14–103 years	Prevalence of moderate-severe and severe periodontitis	(FM)MB-B-DB, (FM)MB-B-DL, RHM, (HM)MB-B-DB, and (HM)MB-B-DL	Not clear	Defined periodontitis at least at 1 site with CAL
Dowsett et al., 2002	Untreated population, randomly selected or from siblings and spouse pairs of the randomly selected subjects, Guatemala	292 subjects, 18–78 years	Prevalence of moderate-severe and severe periodontitis	RHM and Ramfjord teeth	Not clear	Did not state what the minimum number of sites considered to define subjects with periodontitis was; there were untreated subjects, which may reduce the external validity; did not describe the selection criteria for the study population

CDC/AAP: Centers for Disease Control and Prevention and American Academy of Periodontology, CAL: clinical attachment loss, HbA1c: glycated hemoglobin, ARIC: Atherosclerosis Risk in Communities, NHANES: National Health and Nutrition Examination Survey, MB: mesiobuccal, B: midbuccal, DB: distobuccal, DL: distolingual, FM: full-mouth, HM: half-mouth, RHM: random-half-mouth, CPITN: Community Periodontal Index of Treatment Needs, 84 RSSM: 84 sites selected using random site selection method, 42 RSSM: 42 sites selected using random site selection method, and 36 RSSM: 36 sites selected using random site selection method.

**Table 3:** Periodontitis prevalence using partial-mouth recording protocol (PRP) versus full-mouth recording protocol (FRP) at different minimum numbers of sites and disease thresholds defined by clinical attachment loss (CAL) and periodontal probing depth (PPD). Accuracy of the periodontitis prevalence using PRP is reported including the sensitivity, specificity, positive predictive value (PPV), negative predictive value (NPV), and the absolute bias (AB).

Study	Selected PRP, total sites	Minimal interproximal sites with CAL and/or PPD using PRP	Minimal interproximal sites with CAL and/or PPD using FRP	Prevalence PRP (%)	Prevalence FRP (%)	Sensitivity (%)	Specificity (%)	PPV (%)	NPV (%)	AB (%)
Teixeira 2020 [29]	(FM)MB-B-DL Total sites: 84	CAL ≥4 mm, ≥2 site and PPD ≥4 mm, ≥2 site	CAL ≥4 mm, ≥2 site and PPD ≥4 mm, ≥2 site	13.1	18.8	69.7	100.0	100.0	93.4	−5.7
		CAL ≥4 mm, ≥1 site and PPD ≥4 mm, ≥1 site	CAL ≥4 mm, ≥1 site and PPD ≥4 mm, ≥1 site	17.7	22.0	80.5	100.0	100.0	94.8	−4.3
		CAL ≥4 mm, ≥1 site or PPD ≥4 mm, ≥1 site	CAL ≥4 mm, ≥1 site or PPD ≥4 mm, ≥1 site	30.6	34.8	87.7	100.0	100.0	93.8	−4.2
	RHM Total sites: 84	CAL ≥4 mm, ≥2 site and PPD ≥4 mm, ≥2 site	CAL ≥4 mm, ≥2 site and PPD ≥4 mm, ≥2 site	10.6	18.8	56.1	100.0	100.0	90.7	−8.2
		CAL ≥4 mm, ≥1 site and PPD ≥4 mm, ≥1 site	CAL ≥4 mm, ≥1 site and PPD ≥4 mm, ≥1 site	14.3	22.0	64.9	100.0	100.0	91.0	−7.7
		CAL ≥4 mm, ≥1 site or PPD ≥4 mm, ≥1 site	CAL ≥4 mm, ≥1 site or PPD ≥4 mm, ≥1 site	27.1	34.8	77.9	100.0	100.0	89.4	−7.7
	(HM)MB-B-DL Total sites: 42	CAL ≥4 mm, ≥1 site or PPD ≥4 mm, ≥1 site	CAL ≥4 mm, ≥1 site or PPD ≥4 mm, ≥1 site	22.4	34.8	63.9	100.0	100.0	83.8	−12.4
		CAL ≥4 mm, ≥1 site and PPD ≥4 mm, ≥1 site	CAL ≥4 mm, ≥1 site and PPD ≥4 mm, ≥1 site	10.8	22.0	49.4	100.0	100.0	87.5	11.2
		CAL ≥4 mm, ≥2 site and PPD ≥4 mm, ≥2 site	CAL ≥4 mm, ≥2 site and PPD ≥4 mm, ≥2 site	8.3	18.8	43.9	100.0	100.0	88.5	−10.5

Heaton*∗* 2018 [30]	RHM Total sites: 28	CAL ≥6 mm, ≥2 sites and PPD ≥5 mm, ≥1 site	CAL ≥6 mm, ≥2 sites and PPD ≥5 mm, ≥1 site	9.4	18.1	54.1	100.0	100.0	90.8	−8.7
		CAL ≥6 mm, ≥1 site and PPD ≥5 mm, ≥1 site	CAL ≥6 mm, ≥2 sites and PPD ≥5 mm, ≥1 site	13.9	18.1	76.7	95.8	80.2	94.9	−4.2
		CAL ≥6 mm, ≥2 site	CAL ≥6 mm, ≥2 sites and PPD ≥5 mm, ≥1 site	17.0	18.1	94.0	79.6	50.5	98.3	−1.1

Tran*∗* 2014 [10]	(FM)MB-DL Total sites: 56	CAL ≥4 mm, ≥2 sites or PPD ≥5 mm, ≥2 sites	CAL ≥4 mm, ≥2 sites or PPD ≥5 mm, ≥2 sites	30.5	34.3	88.9	100.0	100.0	94.5	−3.8
		CAL ≥4 mm, ≥1 site or PPD ≥5 mm, ≥1 site	CAL ≥4 mm, ≥2 sites or PPD ≥5 mm, ≥2 sites	39.1	34.3	100.0	92.5	87.4	100.0	4.9
		CAL ≥6 mm, ≥2 sites and PPD ≥5 mm, ≥1 site	CAL ≥6 mm, ≥2 sites and PPD ≥5 mm, ≥1 site	8.6	11.8	72.8	100.0	100.0	96.5	−3.2
		CAL ≥6 mm, ≥1 site and PPD ≥5 mm, ≥1 site	CAL ≥6 mm, ≥2 sites and PPD ≥5 mm, ≥1 site	11.5	11.8	100.0	99.3	95.2	100.0	−0.3
	RHM Total sites: 56	CAL ≥4 mm, ≥2 sites or PPD ≥5 mm, ≥2 sites	CAL ≥4 mm, ≥2 sites or PPD ≥5 mm, ≥2 sites	28.1	34.3	81.9	100.0	100.0	91.4	−6.2
		CAL ≥4 mm, ≥1 site or PPD ≥5 mm, ≥1 site	CAL ≥4 mm, ≥2 sites or PPD ≥5 mm, ≥2 sites	37.5	34.2	100.0	95.0	91.2	100.0	3.3
		CAL ≥6 mm, ≥2 sites and PPD ≥5 mm, ≥1 site	CAL ≥6 mm, ≥2 sites and PPD ≥5 mm, ≥1 site	7.7	11.8	65.4	100.0	100.0	95.6	−4.1
		CAL ≥6 mm, ≥1 site and PPD ≥5 mm, ≥1 site	CAL ≥6 mm, ≥2 sites and PPD ≥5 mm, ≥1 site	10.6	11.8	89.8	100.0	100.0	98.7	−1.2

Agerholm and Ashley 1996 [31]	CPITN Total sites: 40	CAL ≥3 mm, ≥2 site	CAL ≥3 mm, ≥2 site	28.7	36.1	79.5	100.0	100.0	89.6	−7.4
		CAL ≥3 mm, ≥1 site	CAL ≥3 mm, ≥1 site	47.5	53.0	89.7	100.0	100.0	89.6	−5.5
		CAL ≥5 mm, ≥2 site	CAL ≥5 mm, ≥2 site	5.9	6.9	85.7	100.0	100.0	99.0	−1.0
		CAL ≥5 mm, ≥1 site	CAL ≥5 mm, ≥1 site	13.3	14.4	93.1	100.0	100.0	98.9	−1.1

FM: full-mouth, (HM): half-mouth, MB: mesiobuccal, B: midbuccal, DB: distobuccal sites, RHM: random-half-mouth, CPITN: Community Periodontal Index of Treatment Needs. *∗* Studies used case definitions by the Centers for Disease Control and Prevention and the American Academy of Periodontology (AAP/CDC), 2007 [18, 19].

**Table 4:** Summary of diabetes mellitus, obesity, and current cigarette smoking associations with periodontal disease defined using the case definitions by the Centers for Disease Control and Prevention and American Academy of Periodontology (CDC/AAP) case definitions.

			Alawaji 2021^1^		Alshihayb 2019^2^		Akinkugbe 2015^3^
			Moderate-severe	Severe	Moderate	Severe	Moderate-severe
Diabetes mellitus	FRP (reference)	OR (95%CI)	0.9 (0.3, 2.7)	2.0 (1.1, 3.7)	1.3 (1.1,1.5)	1.0 (0.7, 1.5)	1.4 (1.2, 1.6)
	42 RSSM	OR (95%CI)					1.3 (1.1, 1.6)
		AB (RB)					0.0 (−0.1)
	RHM	OR (95%CI)	1.6 (0.6, 4.5)	1.9 (0.9, 3.7)	1.2 (1.0, 1.3)	1.0 (0.8, 1.1)	
		AB (RB)	0.6 (−5.0)	−0.1 (−0.1)	−0.1 (−0.4)	−0.1 (−1.8)	
	(FM)MB-DL	OR (95%CI)	1.5 (0.5, 4.0)	1.6 (0.8, 3.0)			
		AB (RB)	0.5 (−4.2)	−0.2 (−0.3)			
	(HM)MB-B-DL	OR (95%CI)					1.2 (1.0, 1.4)
		AB (RB)					−0.1 (−0.4)
	(HM)MB-DL	OR (95%CI)	1.9 (0.8, 4.5)	2.6 (1.3, 5.1)			
		AB (RB)	0.8 (−6.5)	0.3 (0.4)			
	CPITN teeth	OR (95%CI)			1.2 (1.0, 1.5)	1.1 (0.8, 1.6)	
		AB (RB)			−0.1 (−0.3)	0.1 (−2.3)	
	Ramfjord teeth	OR (95%CI)			1.2 (0.8, 1.6)	1.0 (0.6, 1.6)	1.2 (1.0, 1.4)
		AB (RB)			−0.1 (−0.4)	−0.1 (−2.0)	−0.1 (−0.5)

Obesity	FRP (reference)	OR (95% CI)	1.6 (0.7, 3.7)	1.3 (0.7, 2.4)			1.2 (1.1, 1.4)
	42 RSSM	OR (95%CI)					1.2 (1.0, 1.4)
		AB (RB)					−0.1 (−0.3)
	RHM	OR (95%CI)	1.6 (0.8, 3.3)	1.1 (0.5, 2.0)			
		AB (RB)	0.0 (0.0)	−0.2 (−0.8)			
	(FM)MB-DL	OR (95%CI)	1.4 (0.7, 2.9)	1.4 (0.7, 2.6)			
		AB (RB)	−0.1 (−0.2)	0.1 (0.3)			
	(HM)MB-B-DL	OR (95%CI)					1.2 (1.0, 1.4)
		AB (RB)					−0.1 (−0.3)
	(HM)MB-DL	OR (95%CI)	1.1 (0.6, 2.0)	0.8 (0.4, 1.7)			
		AB (RB)	−0.4 (−0.9)	−0.5 (−1.9)			
	Ramfjord teeth	OR (95%CI)					1.1 (0.9, 1.3)
		AB (RB)					−0.2 (−0.7)

Current cigarette smoking	FRP (reference)	OR (95% CI)	4.2 (1.5, 11.7)	2.3 (1.2, 4.7)			3.4 (2.8, 4.1)
	42 RSSM	OR (95%CI)					3.3 (2.7, 3.9)
		AB (RB)					0.9 (2.7)
	RHM	OR (95%CI)	4.0 (1.6, 10.0)	2.5 (1.2, 5.2)			
		AB (RB)	−0.1 (0.0)	0.1 (0.1)			
	(FM)MB-DL	OR (95%CI)	3.5 (1.5, 8.5)	2.2 (1.1, 4.4)			
		AB (RB)	−0.2 (−0.1)	−0.1 (−0.1)			
	(HM)MB-B-DL	OR (95%CI)					3.5 (2.9, 4.2)
		AB (RB)					0.9 (2.9)
	(HM)MB-DL	OR (95%CI)	4.1 (1.9, 9.1)	1.9 (0.9, 4.1)			
		AB (RB)	−0.1 (0.0)	−0.2 (−0.2)			
	Ramfjord teeth	OR (95%CI)					3.2 (2.6, 3.9)
		AB (RB)					1.9 (2.7)

FRP: full-mouth recording protocol, OR: odds ratio, CI: confidence interval, AB: absolute bias, RB: relative bias, 42 RSSM: 42 sites using random site selection method, RHM: random-half-mouth, FM: full-mouth, HM: half-mouth, MB: mesiobuccal, B: midbuccal, DL: distolingual, and CPITN: Community Periodontal Index of Treatment Needs. 1. Analyses adjusted for age, sex, level of education, monthly income, diabetes mellitus (for smoking), smoking (for diabetes mellitus), obesity, perceived stress, and perceived social support. 2. Analyses adjusted for age, gender, race/ethnicity, education, smoking, waist to height ratio, and diabetes mellitus. 3. Analyses adjusted for age, study site, race, gender, education, tooth brushing frequency, frequency of dental visits, and number of teeth present in each selection.

## Data Availability

The data that support the findings of this study are available upon request from the corresponding author.

## Supplementary Materials

Supplementary Materials Appendix Table A-1: list of excluded studies from the systematic review and the reasons for exclusion. Supplementary figures and tables which were referred to in the result section are listed in a separate document.

## References

[1] Lilienfeld D. E. (1978). Definitions of epidemiology. *American Journal of Epidemiology*.

[2] Holtfreter B., Albandar J. M., Dietrich T. (2015). Standards for reporting chronic periodontitis prevalence and severity in epidemiologic studies: proposed standards from the Joint EU/USA Periodontal Epidemiology Working Group. *Journal of Clinical Periodontology*.

[3] Eke P. I., Dye B. A., Wei L. (2015). Update on prevalence of periodontitis in adults in the United States: NHANES 2009 to 2012. *Journal of Periodontology*.

[4] Beck J. D., Caplan D. J., Preisser J. S., Moss K. (2006). Reducing the bias of probing depth and attachment level estimates using random partial-mouth recording. *Community Dentistry and Oral Epidemiology*.

[5] Eke P. I., Thornton-Evans G. O., Wei L., Borgnakke W. S., Dye B. A. (2010). Accuracy of NHANES periodontal examination protocols. *Journal of Dental Research*.

[6] Kingman A., Morrison E., Löe H., Smith J. (1988). Systematic errors in estimating prevalence and severity of periodontal disease. *Journal of Periodontology*.

[7] Susin C., Kingman A., Albandar J. M. (2005). Effect of partial recording protocols on estimates of prevalence of periodontal disease. *Journal of Periodontology*.

[8] Kingman A., Susin C., Albandar J. M. (2008). Effect of partial recording protocols on severity estimates of periodontal disease. *Journal of Clinical Periodontology*.

[9] Tran D. T., Gay I. C., Du X. L. (2016). Partial‐mouth periodontal examination protocol for estimating periodontitis extent and severity in a US population. *Clinical and Experimental Dental Research*.

[10] Tran D. T., Gay I., Du X. L. (2014). Assessment of partial-mouth periodontal examination protocols for periodontitis surveillance. *Journal of Clinical Periodontology*.

[11] Hunt R. J. (1987). The efficiency of half-mouth examinations in estimating the prevalence of periodontal disease. *Journal of Dental Research*.

[12] Hunt R. J., Fann S. J. (1991). Effect of examining half the teeth in a partial periodontal recording of older adults. *Journal of Dental Research*.

[13] Akinkugbe A. A., Saraiya V. M., Preisser J. S., Offenbacher S., Beck J. D. (2015). Bias in estimating the cross-sectional smoking, alcohol, obesity and diabetes associations with moderate-severe periodontitis in the Atherosclerosis Risk in Communities study: comparison of full versus partial-mouth estimates. *Journal of Clinical Periodontology*.

[14] Alshihayb T. S., Kaye E. A., Zhao Y., Leone C. W., Heaton B. (2020). The impact of periodontitis exposure misclassification bias from partial‐mouth measurements on association with diabetes and cardiovascular disease. *Journal of Clinical Periodontology*.

[15] Tran D. T., Gay I., Du X. L. (2013). Assessing periodontitis in populations: a systematic review of the validity of partial-mouth examination protocols. *Journal of Clinical Periodontology*.

[16] Salameh J. P., Bossuyt P. M., McGrath T. A. (2020). Preferred reporting items for systematic review and meta-analysis of diagnostic test accuracy studies (PRISMA-DTA): explanation, elaboration, and checklist. *BMJ*.

[17] Alawaji Y., Alshammari A., Carvalho R., Mostafa N., Aleksejuniene J. (2021). Accuracy of estimating periodontitis and its risk association using partial-mouth recordings for surveillance studies. *A systematic review and meta-analysis*.

[18] Page R., Eke P. (2007). Case definitions for use in population-based surveillance of periodontitis. *Journal of Periodontology*.

[19] Eke P. I., Page R. C., Wei L., Thornton-Evans G., Genco R. J. (2012). Update of the case definitions for population-based surveillance of periodontitis. *Journal of Periodontology*.

[20] Microsoft Corporation (2018). Microsoft Excel. https://office.microsoft.com/excel.

[21] Whiting P. F. (2011). QUADAS-2: a revised tool for the quality assessment of diagnostic accuracy studies. *Annals of Internal Medicine*.

[22] Wallace B. C., Dahabreh I., Trikalinos T., Lau J., Trow P., Schmid C. H. (2012). OpenMetaAnalyst, closing the gap between methodologist and end-Users: R as a computational back-end. *Journal of Statistical Software*.

[23] Deeks J. J., Higgins J. P. T., Altman D. G. (2021). *Cochrane Handbook for Systematic Reviews of Interventions*.

[24] Relvas M., Tomás I., Salazar F., Velazco C., Blanco J., Diz P. (2014). Reliability of partial-mouth recording systems to determine periodontal status: a pilot study in an adult Portuguese population. *Journal of Periodontology*.

[25] Chu Y., Ouyang X. (2015). Accuracy of partial-mouth examination protocols for extent and severity estimates of periodontitis: a study in a Chinese population with chronic periodontitis. *Journal of Periodontology*.

[26] Vettore M. V., Lamarca G. d. A., Leão A. T. T., Sheiham A., Leal M. d. C. (2007). Partial recording protocols for periodontal disease assessment in epidemiological surveys. *Cadernos de Saúde Pública*.

[27] Dowsett S. A., Eckert G. J., Kowolik M. J. (2002). The applicability of half-mouth examination to periodontal disease assessment in untreated adult populations. *Journal of Periodontology*.

[28] Alawaji Y. N., Mostafa N., Carvalho R. M., Alshammari A., Aleksejuniene J. (2022). Accuracy and precision of using partial-mouth recordings to study the prevalence, extent and risk associations of untreated periodontitis. *The Saudi Dental Journal*.

[29] Teixeira F. C. F., Leon L. M., Gomes E. P., Pedrao A. M. N., Costa Pereira A. d., Francisco P. M. S. B. (2020). Comparative evaluation of indices and partial-mouth periodontal protocols for epidemiological surveys. *Journal of Dental Health, Oral Disorders & Therapy*.

[30] Heaton B., Sharma P., Garcia R. I., Dietrich T. (2018). Evaluating periodontal disease misclassification mechanisms under partial-mouth recording protocols. *Journal of Clinical Periodontology*.

[31] Agerholm D. M., Ashley F. P. (1996). Clinical assessment of periodontitis in young adults - evaluation of probing depth and partial recording methods. *Community Dentistry and Oral Epidemiology*.

[32] Preisser J. S., Sanders A. E., Lyles R. H. (2018). Differential misclassification of disease under partial-mouth sampling. *JDR Clinical & Translational Research*.

[33] Nelson D., Holtzman D., Bolen J., Mack K. (2001). Reliability and validity of measures from the behavioral risk factor surveillance system (BRFSS). *Soz Praventivmed*.

[34] Tenny S., Hoffman M. R. (2021). *Prevalence*.

[35] Chapple I. L. C., Genco R. (2013). Workshop working group 2 of the joint E. Diabetes and periodontal diseases: consensus report of the Joint EFP/AAP workshop on periodontitis and systemic diseases. *Journal of Clinical Periodontology*.

[36] Albandar J. M., Susin C., Hughes F. J. (2018). Manifestations of systemic diseases and conditions that affect the periodontal attachment apparatus: case definitions and diagnostic considerations. *Journal of Periodontology*.

[37] Lalla E., Lamster I. B., Schmidt A. M. (1998). Enhanced interaction of advanced glycation end products with their cellular receptor RAGE: implications for the pathogenesis of accelerated periodontal disease in diabetes. *Annals of Periodontology*.

[38] Eaton K. A., Duffy S., Griffiths G. S., Gilthorpe M. S., Johnson N. W. (2001). The influence of partial and full mouth recordings on estimates of prevalence and extent of lifetime cumulative attachment loss: a study in a population of young male military recruits. *Journal of Periodontology*.

[39] Romano F., Perotto S., Castiglione A., Aimetti M. (2019). Prevalence of periodontitis: misclassification, under-recognition or over-diagnosis using partial and full-mouth periodontal examination protocols. *Acta Odontologica Scandinavica*.

[40] Leroy R., Eaton K., Savage A. (2010). Methodological issues in epidemiological studies of periodontitis - how can it be improved?. *BMC Oral Health*.

[41] Beltrán-Aguilar E. D., Eke P. I., Thornton-Evans G., Petersen P. E. (2012). Recording and surveillance systems for periodontal diseases. *Periodontology*.

[42] Botelho J., Machado V., Proença L., Mendes J. J. (2020). The 2018 periodontitis case definition improves accuracy performance of full-mouth partial diagnostic protocols. *Scientific Reports*.

[43] Nomura Y., Morozumi T., Fukuda M. (2020). Optimal examination sites for periodontal disease evaluation: applying the item response theory graded response model. *Journal of Clinical Medicine*.

[44] Dye B. A. (2000). Global periodontal disease epidemiology: global periodontal disease epidemiology. *Periodontology*.

