# Supplementary material for: Accuracy of Estimating Periodontitis and Its Risk Association Using Partial-Mouth Recordings for Surveillance Studies: A Systematic Review and Meta-Analysis
Source: Int J Dent. 2022 Mar 17;2022:7961199. doi: 10.1155/2022/7961199 (PMC8947864; doi:10.1155/2022/7961199)
Supplement: Supplementary Materials — Appendix Table A-1: list of excluded studies from the systematic review and the reasons for exclusion. Supplementary figures and tables which were referred to in the result section are listed in a separate document. [file 7961199.f1.zip › 7961199.f1/Appendix - Exluded studies (2).docx]

**Appendix**

**Table A-1.** List of excluded studies from the systematic review and the reasons for exclusion

| **First author, date** | **Reasons for exclusion** |  |
| --- | --- | --- |
| **Alshihayb, 2021** | Simulation study. |  |
| **Numora, 2020** | Did not report periodontal outcomes. |  |
| **Botelho, 2020** | Did not report periodontal outcomes and used the National Health And Nutrition Examination Survey (NHANES) dataset that was utilized earlier in other included studies (8,11,90) |  |
| **Heaton, 2018** | Reported the sensitivity for diagnosing severe cases based on mesiobuccal and distolingual sites only. The severity estimate was provided as mean CAL without reporting the SD. |  |
| **Preisser, 2018** | Simulation study. |  |
| **Heaton, 2018** | Simulation study. |  |
| **Machado, 2017** | Examined the gingivitis only. |  |
| **Preisser, 2017** | The Atherosclerosis Risk in Communities (ARIC) dataset, periodontal outcomes and PRP used in this study were similar to what were reported earlier in Beck's study but used different statistical approach to estimate the prevalence using conditional linear family estimator then compared it to standard estimator. |  |
| **Peres, 2013** | Assessed bleeding on probing, calculus, and PPD. No assessment for CAL. |  |
| **Eke, 2010** | The FRP included data measured using PRP. |  |
| **Bassani, 2006** | Used CPITN without assessment of CAL. |  |
| **Borges Yane, 2004** | FRP excluded the sites included in the PRP. Thus, the mesiobuccal sites of all teeth were not considered as part of the gold standard. |  |
| **Mumghamba, 2004** | Used Ramfjord index. |  |
| **Owens, 2003** | Reported the prevalence and estimates of severity and extent for full mouth only. The estimate of severity (mean CAL) of partial mouth recording was reported without reporting the SD. The number of subjects with disease or without disease for full mouth and partial mouth recordings were not provided. |  |
| **Thomson, 2002** | Assessed full mouth at three sites per tooth only. |  |
| **Eaton, 2001** | Assessed the CAL at different thresholds (3mm or less). |  |
| **Benigeri, 2000** | Used CPITN index. |  |
| **Agerholm, 1996** | Assessed the CAL at different thresholds (3mm or 5mm). |  |
| **Baelum, 1993** | Used CPITN index. |  |
| **Papapanou, 1993** | Used ESI index and did not report the prevalence or made information at the individual level, also it did not report the mean (SD) for extent and severity for partial mouth. |  |
| **Diamanti-Kipioti, 1993** | Half mouth was assessed at two sites only, no CAL was reported. Full mouth was assessed at our sites including midbuccal and midlingual. |  |
| **Rams, 1993** | Examined the periodontal disease progression using CPITN without reporting the CAL. |  |
| **Almas, 1991** | Did not assess the CAL. |  |
| **Hunt, 1991** | Used CPITN and full mouth assessments at two sites only in some of the study subjects. |  |
| **Silness, 1988** | Assessed full mouth only at three sites per tooth. |  |
| **Kingman, 1988** | Assessed full mouth at four sites (not restricted to interproximal sites) per tooth. |  |
| **Hunt, 1987** | Used CPITN without assessment of CAL. |  |
| **Fleiss, 1987** | Used CPITN without assessment of CAL. |  |
| **Goldberg, 1985** | Examined the gingivitis and plaque in children and young adults. |  |
| **Gettinger, 1982** | Assessed full mouth only at two sites per tooth. |  |
| **Mills, 1975** | Assessed the correlation between PRP and FRP without reporting the number of subjects, prevalence or estimates of severity for PRP versus FRP. |  |
| **PRP:** Partial mouth recording, **FRP:** Full mouth recording**, SD:** standard deviation, **PPD:** Periodontal Probing Depth, **CAL:** Clinical Attachment Loss. **ESI:** Extent and Severity Index, **CPITN:** Community Periodontal Index of Treatment Need. | | |
