# Supplementary material for: Accuracy of Estimating Periodontitis and Its Risk Association Using Partial-Mouth Recordings for Surveillance Studies: A Systematic Review and Meta-Analysis
Source: Int J Dent. 2022 Mar 17;2022:7961199. doi: 10.1155/2022/7961199 (PMC8947864; doi:10.1155/2022/7961199)
Supplement: Supplementary Materials — Appendix Table A-1: list of excluded studies from the systematic review and the reasons for exclusion. Supplementary figures and tables which were referred to in the result section are listed in a separate document. [file 7961199.f1.zip › 7961199.f1/supplemetary materials (2).docx]

**Figure S-1.** Summary of the Absolute Bias **(AB)**_prevalence_ of CDC/AAP severe periodontitis using Partial-mouth Recording Protocols **(PRP)**. Total PRP sites for each PRP are listed. AB_prevalence_ values <0.0 underestimate the prevalence while values >0.0 overestimate it.

**FRP:** Full-mouth Recording Protocol, **N:** sample size, **CI**: Confidence Interval**, MB:** Mesio-buccal, **B:** Mid-buccal, **DB:** Disto-buccal, **ML:** Mesio-lingual, **L:** Mid-lingual, **DL:** Disto-lingual, **(FM):** Full-mouth, **(HM):** Half-mouth, **42 RSSM:** 42 sites selected using Random-Site-Selection-Method**, (RHM):** Random-Half-mouth, **CPITN:** Community Periodontal Index of Treatment Needs.

**Figure S-2.** Summary of the sensitivity of CDC/AAP moderate-severe periodontitis prevalence using Partial-mouth Recording Protocols **(PRP)**. Specificity and positive predictive value are 100% for all PRPs. PRP sites, minimal number of sites with Clinical Attachment Loss **(CAL)** and Negative Predictive Value **(NPV)** are listed.

**FRP:** Full-mouth Recording Protocol, **N:** sample size, **CI**: Confidence Interval**, MB:** Mesio-buccal, **B:** Mid-buccal, **DB:** Disto-buccal, **ML:** Mesio-lingual, **L:** Mid-lingual, **DL:** Disto-lingual, **(FM):** Full-mouth, **(HM):** Half-mouth, **42 RSSM:** 42 sites selected using Random-Site-Selection-Method**, (RHM):** Random-Half-mouth, **CPITN:** Community Periodontal Index of Treatment Needs.

**
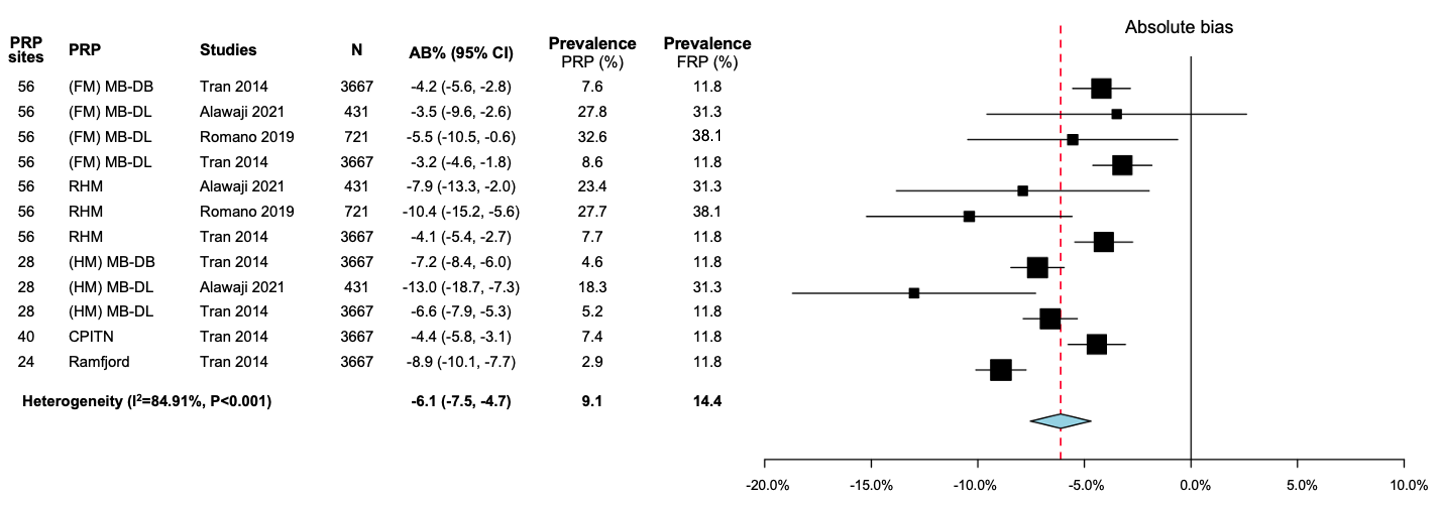
**

**Figure S-3.** Summary of the sensitivity of CDC/AAP severe periodontitis prevalence using Partial-mouth Recording Protocols **(PRP)**. Specificity and positive predictive value are 100% for all PRPs. PRP sites, minimal number of sites with Clinical Attachment Loss (CAL) and Negative Predictive Value **(NPV)** are listed.

**FRP:** Full-mouth Recording Protocol, **N:** sample size, **CI**: Confidence Interval**, MB:** Mesio-buccal, **B:** Mid-buccal, **DB:** Disto-buccal, **ML:** Mesio-lingual, **L:** Mid-lingual, **DL:** Disto-lingual, **(FM):** Full-mouth, **(HM):** Half-mouth, **(RHM):** Random half-mouth, **CPITN:** Community Periodontal Index of Treatment Needs, **?:** not clear.


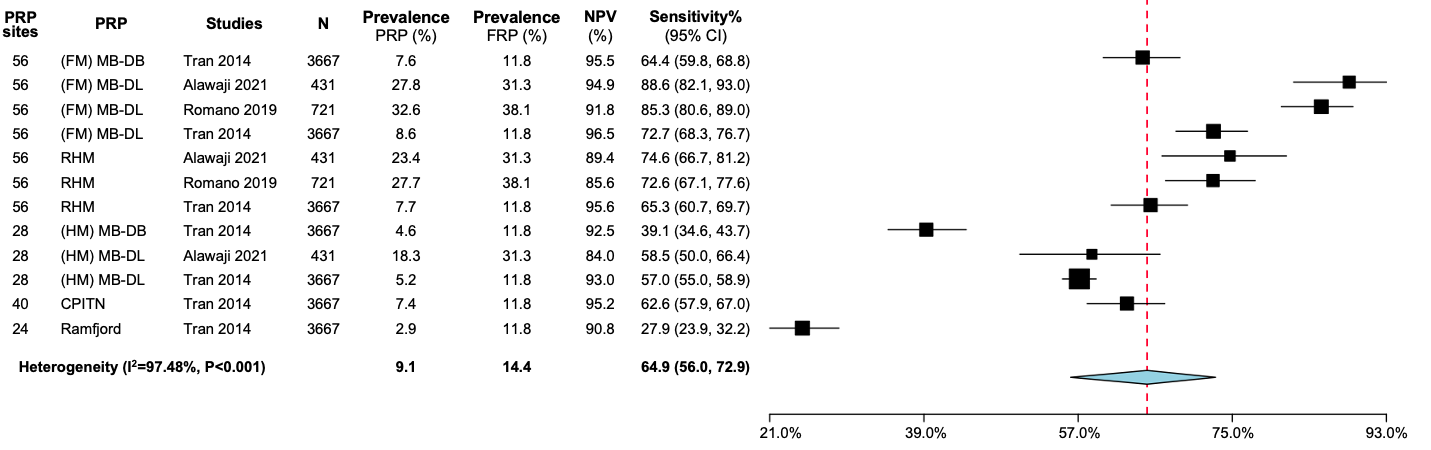


**Figure S-4.** Summary of sensitivity of CDC/AAP severe periodontitis. Background characteristics of studies, PRP sites and specific thresholds of Clinical Attachment Loss **(CAL)** are listed.

**FRP:** Full-mouth Recording, Protocol, **N:** sample size, **CI**: Confidence Interval**, NHANES:** National Health And Nutrition Examination Survey, **MB:** Mesio-Buccal, **B:** Mid-Buccal, **DB:** Disto-Buccal, **ML:** Mesio-Lingual, **L:** Mid-Lingual, **DL:** Disto-Lingual, **(FM):** Full-Mouth, **(HM):** Half-Mouth, **CPITN:** Community Periodontal Index of Treatment Needs.

**Table S-1.** Customization of the QUADAS-2 tool for quality assessment of diagnostic studies

| **Domain** | **Quality assessment** | **Original signaling question** | **Customized signaling questions for the current review** |
| --- | --- | --- | --- |
| 1. **Patient selection** | Risk of bias | Could the selection of patients have introduced bias? | Did the study use consecutive or random sample? |
|  |  | Was a case control design avoided? | Excluded question. |
|  |  | Did the study avoid inappropriate exclusions? | Did the study have clear eligibility criteria, description of baseline characteristics, study settings, and avoided any inappropriate exclusion? |
|  | Applicability concerns | Are there concerns that the included patients and setting do not match the review question? | Were the spectrum of subjects, representative of the subjects who will receive the examination in the general population? |
|  |  |  | Added question: Did they include subjects with few remaining teeth? (Studies that excluded subjects with >6 teeth were considered to have high applicability concerns). |
| 1. **Index test: Partial-mouth Recording Protocol (PRP)** | Risk of bias | Were the index test results interpreted without knowledge of the results of the reference standard? | Excluded question |
|  |  | If a threshold was used, was it prespecified? | Did the study adequately define the diagnostic thresholds for periodontitis and provided a rationale for using certain diagnostic thresholds? |
|  | Applicability concerns | Are there concerns that the index test, its conduct, or its interpretation differ from the review question? | Is there a concern regarding the PRP selection, use or interpretation that differed from the review question? |
| 1. **Reference standard: Full-mouth Recording Protocol (FRP)** | Risk of bias | Is the reference standard likely to correctly classify the target condition? | Is the reference standard likely to correctly classify the subjects with periodontitis? |
|  |  | Were the reference standard results interpreted without knowledge of the results of the index test? | Excluded question |
|  | Applicability concerns | Are there concerns that the target condition as defined by the reference standard does not match the question? | Could the reference standard, its conduct or interpretation have deviated from the review question? |
| 1. **Flow and Timing** | Risk of bias | Was there an appropriate interval between the index test and reference standard? | Excluded question |
|  |  | Did all patients receive the same reference standard? | Did all the patients receive the same FRP examination? |
|  |  | Were all patients included in the analysis? | Excluded question |
|  |  |  | Added question: Were the clinical examinations conducted by a trained and calibrated examiner/s? |
|  |  |  | Added question: Were there any intra-examiner/ inter-examiner reliability tests reported? |
| **QUADAS-2**: 2^nd^ version of QUALITY Assessment of Diagnostic accuracy studies**, FRP**: Full-mouth Recording Protocol, **PRP:** Partial-mouth Recording Protocol. | | | |
